# AFFORDABILITY, PERCEIVED RACISM, AND HEALTHCARE SYSTEM DISTRUST AMONG AFRICAN AMERICAN WOMEN AGED 45 AND OVER

**DOI:** 10.1093/geroni/igac059.2868

**Published:** 2022-12-20

**Authors:** Jacqueline Wiltshire, Carla Sampson, Keith Elder, Paul Musey

**Affiliations:** Indiana University, Indianapolis, Indiana, United States; New York University, New York, New York, United States; Mississippi, Clinton, Mississippi, United States; Indiana University, Indianapolis, Indiana, United States

## Abstract

Unaffordable healthcare costs—a major concern for Americans--disproportionately affect African Americans (AA), who are more likely to distrust the healthcare system due to past mistreatment and discrimination. However, the relationships between affordability, mistreatment, discrimination, and healthcare system distrust are unclear. Using cross-sectional survey data from a community-based sample of 313 African American women aged 45 and over, we assessed the relationships between the ability to get needed care due to costs, negative healthcare experiences, perceived racism, and healthcare system distrust. Linear regression and mediation analyses were conducted to assess relationships. Approximately 23% of women reported the inability to get needed care because of costs, and 44% had a negative experience with a healthcare provider. Healthcare system distrust was higher among women unable to get healthcare because of costs (β = 2.66; p = 0.005) or had a negative experience with a healthcare provider (β = 3.02; p < 0.001). However, perceived racism in the healthcare system, a significant predictor of distrust (β = 0.81; p < 0.001), attenuated the relationships between inability to get health care because of costs (β = 1.74; p = 0.051) and negative experience with a healthcare provider (β = 1.79; p = 0.013). Perceived racism explains 34% and 46%, respectively, of the relationships between affordability, negative experience with healthcare provider, and healthcare system distrust. These findings are important given the relevance of building trust and understanding needs to address health inequities. Future research should explore whether these findings hold for AA men and other minoritized groups.

